# Bioactive Peptides and Protein Hydrolysates as Lipoxygenase Inhibitors

**DOI:** 10.3390/biology12070917

**Published:** 2023-06-27

**Authors:** Fai-Chu Wong, Tsun-Thai Chai

**Affiliations:** 1 Department of Chemical Science, Faculty of Science, Universiti Tunku Abdul Rahman, Kampar 31900, Malaysia; wongfc@utar.edu.my; 2 Center for Agriculture and Food Research, Universiti Tunku Abdul Rahman, Kampar 31900, Malaysia

**Keywords:** anti-lipoxygenase peptide, enzymatic hydrolysis, inflammation, lipoxygenase inhibitory activity

## Abstract

**Simple Summary:**

This review is about lipoxygenases, which are enzymes present in the human body that cause inflammation when they come into contact with certain fats. Compounds that block the action of lipoxygenases can potentially help stop disease and inflammation. Such compounds can also be used to produce pharmaceuticals, cosmetics, and food products. Bioactive peptides are tiny parts of a protein that can be released when the protein is broken down. These peptides can promote health in many different ways, including blocking the action of lipoxygenases. In this review, peptides from grains, seeds, insects, milk, fish feed, antler blood, fish scales, and feathers are explored. Their activities and they means by which they block the action of lipoxygenases are discussed. Potential research directions for scientists to consider in the future to help discover new peptides that can block the action of lipoxygenases are proposed.

**Abstract:**

Lipoxygenases are non-heme iron-containing enzymes that catalyze the oxidation of polyunsaturated fatty acids, resulting in the production of lipid hydroperoxides, which are precursors of inflammatory lipid mediators. These enzymes are widely distributed in humans, other eukaryotes, and cyanobacteria. Lipoxygenases hold promise as therapeutic targets for several human diseases, including cancer and inflammation-related disorders. Inhibitors of lipoxygenase have potential applications in pharmaceuticals, cosmetics, and food. Bioactive peptides are short amino acid sequences embedded within parent proteins, which can be released by enzymatic hydrolysis, microbial fermentation, and gastrointestinal digestion. A wide variety of bioactivities have been documented for protein hydrolysates and peptides derived from different biological sources. Recent findings indicate that protein hydrolysates and peptides derived from both edible and non-edible bioresources can act as lipoxygenase inhibitors. This review aims to provide an overview of the current knowledge regarding the production of anti-lipoxygenase protein hydrolysates and peptides from millet grains, chia seeds, insects, milk proteins, fish feed, velvet antler blood, fish scales, and feather keratins. The anti-lipoxygenase activities and modes of action of these protein hydrolysates and peptides are discussed. The strengths and shortcomings of previous research in this area are emphasized. Additionally, potential research directions and areas for improvement are suggested to accelerate the discovery of anti-lipoxygenase peptides in the near future.

## 1. Introduction

Bioactive peptides are short fragments ranging between 2 and 20 residues that are initially encrypted in an inactive state in a parent protein. Such fragments exhibit their bioactivities after they are released from the parent protein [1,2,3]. To date, a large number of bioactive peptides that are capable of modulating biological functions of the human body and those that can tackle the activity of pathogenic organisms have been documented [4]. Such peptides can exert their effects in a variety of ways, including the inhibition of enzymes associated with metabolic syndrome and inflammation [5,6,7,8], the disruption of protein–protein interactions, the regulation of gene and protein expression, and the modulation of cellular signaling pathways [9,10]. Bioactive peptides could be released from dietary proteins during in vivo gastrointestinal (GI) digestion. They can also be generated from other protein-rich samples by means of enzymatic proteolysis and microbial fermentation [1,2,3,11]. The raw materials that have been documented as sources of bioactive peptides are diverse and numerous [12]. They range from edible materials, such as seafood [2,13], edible insects [14], spices [15], seeds [16], and traditional medicine [17], to non-edible marine organisms, such as the barrel sponge (*Xestospongia testudinaria*) [18]. Additionally, agricultural by-products, such as poultry feathers [19], fish scales [20], and corn silk [21], are also sources of bioactive peptides. 

“Protein hydrolysate” refers to the product of the hydrolytic action of protease(s) on a complex proteinaceous sample or a pure protein sample. Protein hydrolysates are essentially a mixture of free amino acids, peptides, and possibly even partially degraded proteins. Protein hydrolysates are generally regarded as a crude peptide mixture. Owing to the crude nature of a protein hydrolysate, peptides of opposite bioactivity, such as prooxidant peptides vs. antioxidant peptides, may co-exist in the same hydrolysate [22,23]. The presence of non-bioactive peptides or low availability of the bioactive peptides of interest may lead to the detection of poor bioactivity. Protein hydrolysates often serve as the initial raw material for bioactivity testing and subsequently, as the sources from which bioactive peptides can be isolated and identified, facilitated by a series of bioassay- or chemical assay-guided fractionation steps [1,2,3]. 

The past two decades have seen a remarkable growth in research interest in bioactive peptide discovery. Supporting this is the trend of 22 Scopus-indexed publications having “bioactive peptide” as their keyword in the year 2002 rising to 492 publications in the year 2022 (accessed on 29 May 2023). This surge in interest surrounding bioactive peptides is driven by an expanding understanding of their diverse applications, including functional food/beverage development, health benefits, agricultural applications, and their potential contribution to peptide drug discovery. Functional food ingredients developed from the bioactive peptides and protein hydrolysates of whey proteins, with claimed benefits such as inflammation suppression and blood pressure reduction, have been commercially marketed [24]. Fish protein hydrolysates are also marketed worldwide for nutritional, cosmetic, and pharmaceutical applications [25]. The benefits of fish protein hydrolysates as aquaculture feed, which promote fish growth, immunity, and disease resistance, at least in part mediated by the action of bioactive peptides, have been well established [26]. On the other hand, there has been a steady 4-fold increase in the cumulative number of therapeutic peptides approved for markets in the United States, Europe, and/or Japan over the past four decades, with 149 peptides in active clinical development as of May 2021 [27]. Bioactive peptide research is expected to provide potential lead candidates for future peptide drug discovery [28,29]. 

**Figure 1:**
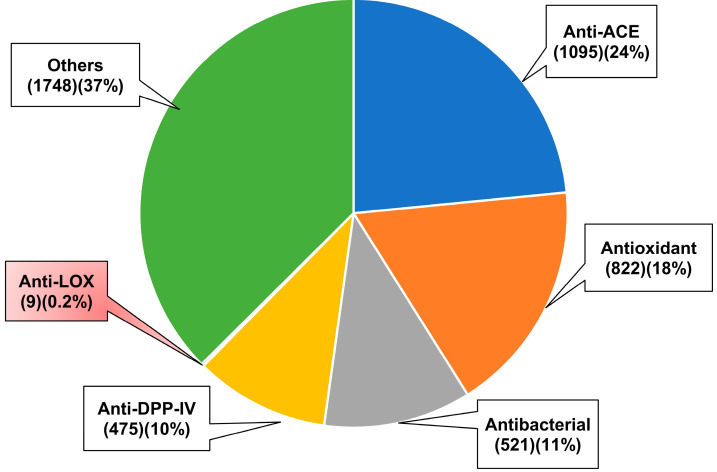
Distribution of major classes of bioactive peptides deposited in the BIOPEP-UWM database (accessed on 27 May 2023). The numbers in the brackets are the number and percentage of the peptides in the given category. ACE, angiotensin-converting enzyme; DPP-IV, dipeptidyl peptidase-IV; LOX, lipoxygenase.

Lipoxygenases (LOX) are a family of non-heme iron-containing enzymes that catalyze the oxidation of polyunsaturated fatty acids, ultimately leading to the production of lipid hydroperoxides. These enzymes are widely distributed in eukaryotes (animals, plants, and fungi) and cyanobacteria. The primary substrates of LOX in plant cells are linoleic and linolenic acids, whereas in animal cells, arachidonic acid is the main substrate [30,31]. LOXs are considered promising therapeutic targets for a number of human diseases, including cancer [30,31] and inflammation-related disorders [32]. Currently, there is an urgent need to discover novel LOX inhibitors as a strategy to combat various human diseases [32]. A search of the BIOPEP-UWM database (https://biochemia.uwm.edu.pl/en/biopep-uwm-2/) (accessed on 27 May 2023) revealed that only 0.2% of the 4670 deposited peptide sequences were validated anti-LOX peptides (Figure 1). In comparison, there are approximately 120 times more peptides with validated anti-angiotensin converting enzyme (anti-ACE) activity; ACE is a therapeutic target for the control of hypertension [4]. Current research appears to have focused less on the identification of anti-LOX peptides compared to other types of bioactive peptides. Many studies have focused on characterizing the anti-LOX potency of protein hydrolysates rather than identifying the anti-LOX peptides present in these hydrolysates, which will be discussed in the sections below. To the best of our knowledge, there is no recent review in the current literature that specifically addresses anti-LOX peptides. Therefore, this review aims to consolidate the recent emerging evidence regarding the anti-LOX properties of protein hydrolysates and peptides derived from various food and non-food sources. The review will provide an overview of the methods used to generate anti-LOX protein hydrolysates, as well as the purification and identification of anti-LOX peptides. Findings on the potency and modes of action of both anti-LOX peptides and protein hydrolysates will be summarized, with emphasis on the peptides. Future research opportunities are highlighted.

## 2. LOX

There are six arachidonate LOXs in humans, including 5-LOX, 12-LOX, and 15-LOX. The genes encoding these enzymes, their tissue distribution, and the products of their action on arachidonic acid have been reviewed [33,34]. The nomenclature of LOX enzymes corresponds to the position of the carbon in the fatty acid that the enzyme oxygenates. For example, human 5-LOX oxygenates carbon 5 on arachidonic acid, converting it to 5-hydroperoxyeicosatetraenoic acid (5-HPETE) [30,31]. It is understood that 5-HPETE may serve as a precursor in the production of proinflammatory lipid mediators in human cells [34]. LOX enzymes have been implicated in the pathogenesis of human diseases, including several cancers, chronic liver disease, atherosclerosis, and asthma [33,34]. Consequently, inhibition of LOX is considered an important strategy for disease prevention and treatment, and LOX inhibitors have attracted considerable attention from the medical community [31,34]. LOX is responsible for many inflammatory skin problems, such as the redness, rashes, or edema characteristic of many skin diseases. Therefore, LOX inhibitors are considered to have skin care or cosmetic applications [35,36,37]. On the other hand, the products of undesired LOX reactions can affect the quality of food. Legumes, which are rich in fatty acids, are particularly susceptible to LOX-associated food spoilage. The action of LOX on unsaturated fatty acids can lead to rancidity, accompanied by the development of off-flavors and odors in legumes and legume-based products. In addition, LOX activity can also affect the color, aroma, and flavor of oil and oil-containing foods during processing and storage [30]. In short, LOX inhibition not only provides a valuable approach to the prevention and treatment of human diseases, but the control of LOX activity is also relevant to the food industry.

## 3. Production of Anti-LOX Protein Hydrolysates and Peptides

The production of anti-LOX protein hydrolysates and bioactive peptides from various biological sources, including edible plant proteins (proso millet and chia seeds) [38,39], edible animal proteins (insects and milk) [40,41], velvet antler blood [17], and agricultural wastes (e.g., poultry feathers and fish scales) [19,42], has been documented. Protein hydrolysis, facilitated primarily by enzymatic hydrolysis and less commonly by microbial degradation, has been used to liberate anti-LOX peptides from biological samples (Table 1). Enzymatic hydrolysis in the form of simulated GI digestion, as mediated by the action of pepsin and pancreatin, has been employed to generate anti-LOX protein hydrolysates and peptides from velvet antler blood [17] and chia seed proteins [39]. In comparison, the simulated GI digestion experiments performed on insect proteins [40] and millet grain protein fractions [38] were more representative of human GI digestion because they also simulated oral digestion by using α-amylase in artificial saliva, in addition to simulating gastric digestion with pepsin, and intestinal digestion with pancreatin and bile extract (Table 1). Simulated GI digestion is an interesting experimental approach because it may reveal the potential benefit of dietary proteins in terms of their ability to release GI-resistant anti-LOX peptides after oral ingestion. GI resistance does not imply GI absorption or uptake. However, GI-resistant anti-LOX peptides remain valuable because they are not susceptible to further degradation, reducing the risk of losing their bioactivity before intestinal absorption can occur. In contrast to the common approach of hydrolyzing protein samples with commercially available proteases, Kshetri and coworkers [19] used locally isolated keratinolytic bacteria, namely *Streptomyces tanashiensis*-RCM-SSR-6 and *Bacillus* sp. RCM-SSR-102 [43,44], to perform microbial hydrolysis of chicken feather waste.

When preparing anti-LOX protein hydrolysates, some researchers prepared protein isolates or fractions from their samples prior to protein hydrolysis [38,39,40], while others did not [19]. Focusing on three insect species (mealworms, locusts, and crickets), Zielińska and coworkers [40] compared the anti-LOX activities of hydrolysates prepared from whole insects and insect protein isolates. They found that hydrolysates of insect proteins (IC_50_ = 0.65–0.89 mg/mL) exhibited a stronger anti-LOX activity than the hydrolysates of whole insects (IC_50_ = 1.30–3.14 mg/mL). Thus, both groups of hydrolysates exerted anti-LOX activity, although the use of insect protein isolates as raw material led to stronger anti-LOX activity [40]. Consequently, the use of protein isolates is not an absolute prerequisite for the production of anti-LOX protein hydrolysates. The possibility of generating an anti-LOX protein hydrolysate without having to isolate proteins may simply workflow, reduce costs, and save time. This may also promote the utilization of anti-LOX protein hydrolysates in the food and cosmetic industry. 

Some researchers use sodium dodecyl sulfate-polyacrylamide gel electrophoresis (SDS-PAGE) to monitor the extent of protein sample hydrolysis and to estimate the molecular weight distribution of the major proteins/peptides in the hydrolyzed samples [17,19,45]. To monitor the extent of protein hydrolysis, specifically the percentage of cleaved peptide bonds, Grancieri and coworkers [39] analyzed the degree of hydrolysis (DH) of chia seed protein fractions after simulated GI digestion. The authors found that the DH of protein hydrolysates did not correlate closely with their anti-LOX activity [39]. This suggests that although DH is useful for monitoring the extent or effectiveness of proteolysis, it is not a reliable indicator of the anti-LOX activity of protein hydrolysates.

The strategy employed by Ding and coworkers [16] for isolating and identifying anti-LOX peptides from velvet antler blood hydrolysate is typical of how numerous other bioactive peptides were discovered in the literature [1,2,3]. Briefly, the authors used a combination of non-chromatographic (membrane ultrafiltration) and chromatographic (gel filtration chromatography) methods to fractionate the hydrolysate, guided by an in vitro anti-LOX assay. The desired gel filtration chromatography fraction was finally subjected to liquid chromatography-tandem mass spectrometry (LC-MS/MS) analysis to identify the peptide sequences present in the fraction. Ding and coworkers [17] identified 219 peptides from a gel filtration chromatographic fraction of velvet antler blood hydrolysate. Synthesis of all 219 peptides for in vitro activity validation would be costly and laborious. Therefore, the authors used in silico screening tools to narrow down the entire set of putative bioactive peptides to eight candidates before synthesizing and testing them for in vitro anti-LOX activity [17]. To our knowledge, there are no in silico tools specifically designed to predict anti-LOX activities of peptide sequences. Therefore, it is not surprising that the in silico prediction tools used by Ding and coworkers [17] in their study were generic and not anti-LOX peptide specific, i.e., PeptideRanker and AntiInflam tools. PeptideRanker [46] predicts the probability that a peptide sequence is generally bioactive, whereas AntiInflam [47] predicts anti-inflammatory peptides. It should be noted that inflammation is not solely regulated by LOX activity. Therefore, anti-inflammatory peptides are not equivalent to anti-LOX peptides. Despite the fact that in silico prediction tools specific for the type of peptide of interest are not always available, such an approach still has its advantages. In fact, the integrated in vitro-in silico approach used by Ding and coworkers [17] for the discovery of anti-LOX peptides has also been adopted by previous studies for the discovery of other bioactive peptides, particularly for the purpose of shortlisting potential candidates from a relatively large set of peptide sequences for further analysis or peptide synthesis [21,48,49,50].

In bioactive peptide discovery, peptide synthesis is the next logical step after the peptide sequence identification. Such a step is crucial because the final purified active fraction isolated by researchers often comprise multiple peptide sequences, some of which may not exert the desired bioactivity. For instance, in our previous work [18], two peptides (KENPVLSLVNGMF and LLATIPKVGVFSILV) were identified from a cytotoxic peptide fraction derived from marine sponge protein hydrolysate. Only the peptide KENPVLSLVNGMF showed cytotoxicity in a dose-dependent manner [18]. In other cases, while all peptide sequences present in an active fraction possessed the desired bioactivity, thus accounting for the overall bioactivity exerted by the active fraction, the peptides may vary in their relative levels of the desired bioactivity [13]. Furthermore, in cases where peptide candidates have been shortlisted using in silico prediction tools, especially tools not specifically designed for the bioactivity under investigation, the validation of the bioactivity of the peptide sequences is highly desirable. Through peptide identification and the bioactivity validation of synthetic peptides, the specific peptide sequences responsible for the bioactivity of the protein hydrolysate and/or purified fractions can be identified. 

In the context of peptide identification, discrepancies between theoretically expected fragments from a hydrolyzed protein and those actually detected from the hydrolysate have been reported. For example, in the search for anti-LOX peptides from β-casein tryptic digest, Rival and coworkers [45] identified a missed cleavage peptide segment (VKEAMAPK). In addition, the authors found a peptide sequence resulting from an unexpected cleavage of the Ser-Lys peptide bond in β-casein by trypsin. The authors suspected that such a result may be related to chymotrypsin activity in the commercial trypsin preparation they used, or even some other “unusual and unexplained” enzyme activity. According to Heissel and coworkers [51], commercial trypsin preparations of the highest purity have no or very low activity of contaminating proteases, but other preparations may have low non-tryptic activity due to the presence of co-purified chymotrypsin. During tryptic hydrolysis, the enzyme may also self-digest, yielding a pseudotrypsin form with chymotryptic activity [51].

In studies of anti-LOX peptides and protein hydrolysates, the soybean LOX has often been used as a model for in vitro LOX inhibition assays using linoleic acid as a substrate [17,40,45,52]. The activity of LOX was determined by monitoring the formation of reaction products at 234 nm [17,39,40] or, less commonly, by monitoring the rate of oxygen consumption during the catalytic reaction [45,52]. Positive control or reference compounds such as nordihydroguaiaretic acid [19], diclofenac sodium [17], and ascorbic acid [39] were used in several studies. Nevertheless, bioinformatic analysis by Cengiz Şahin and Cavas [53] suggested that soybean LOXs are not a suitable model for human LOXs due to significant sequence-based differences. In concurrence with this, Muñoz-Ramírez and coworkers [54] found that although catechols extracted from *Lithraea caustica* inhibited both soybean 15-LOX and human 5-LOX, the catechols were more selective against the human LOX. Furthermore, aqueous infusion of *L. caustica* effectively inhibited human LOX, although it did not inhibit soybean LOX [54]. Therefore, future works on anti-LOX peptides should take into account that inhibition of soybean LOX does not necessarily indicate inhibition of human LOX. Thus, if the goal is to discover anti-LOX peptides for health promotion or disease management, validation of candidate peptide inhibition of human LOX is essential. If feasible, the use of human LOX in the anti-LOX assay-guided purification of peptides is highly recommended. However, if the goal is only to search for anti-LOX peptides or hydrolysates for applications in reducing LOX-mediated food spoilage or other non-human applications, the need to use human LOX in the anti-LOX assay is less critical.

## 4. Potency and Modes of Action

**Figure 2:**
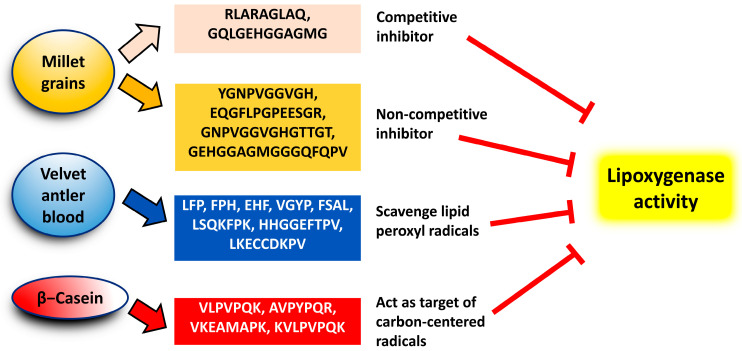
Summary of modes of action of 18 anti-LOX peptides.

Table 2 presents 18 anti-LOX peptides reported in the literature. These peptides range in length from 3 to 16 residues, with molecular masses of approximately up to 1400 Da. Figure 2 depicts a graphical summary of the modes of action proposed for the 18 anti-LOX peptides listed in Table 2.

**Figure 3:**
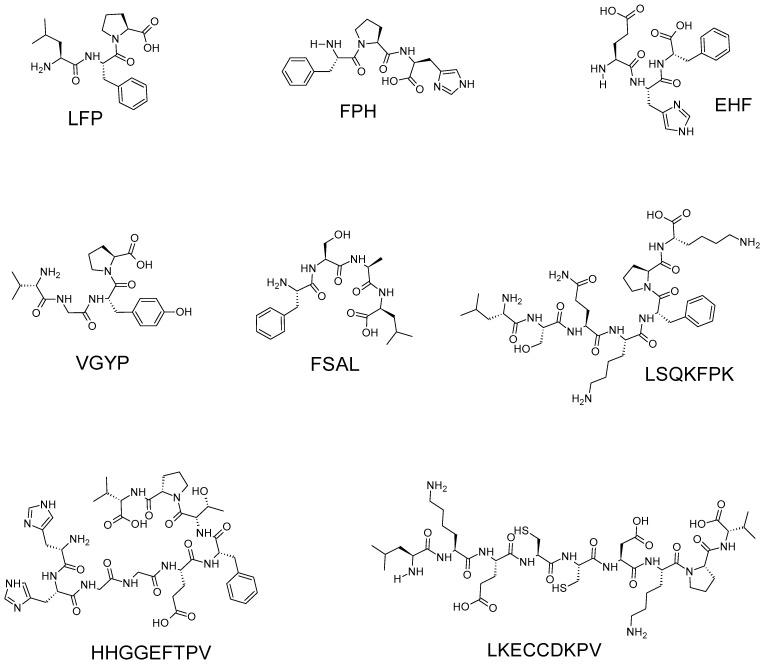
Anti-LOX peptides identified from a velvet antler blood hydrolysate [17]. Peptides were drawn using the ACD/ChemSketch freeware (version 2022.1.0, Advanced Chemistry Development, Inc. (ACD/Labs), Toronto, ON, Canada, www.acdlabs.com).

Eight anti-LOX peptides ranging from three to nine residues were identified from velvet antler blood hydrolysate [17] (Figure 3). The eight peptides were individually less potent (<12% anti-LOX activity) than diclofenac sodium (approximately 85% activity), a commonly prescribed nonsteroidal anti-inflammatory drug that exhibits anti-LOX activity [56]. The peptides were only tested at a single sample concentration (1 mg/mL) and IC_50_ values were not reported. The peptides FSAL and LFP, exhibiting approximately 12 and 10% activity, respectively, were the strongest among the eight peptides. Notably, the release of these peptides during simulated GI digestion implies resistance of the peptides to GI degradation, which at least partially supports the anti-LOX potential of velvet antler blood after oral ingestion [17]. 

Ding and coworkers [17] also reported that the eight peptides all showed weaker anti-LOX activity than the gel filtration chromatographic fraction GF-2 (26%) from which they were isolated. Therefore, the anti-LOX activity of partially purified peptide fraction GF-2 may have resulted from synergism between multiple peptides present in the fraction. GF-2 apparently holds more potential as an anti-LOX agent when compared to the eight individual peptides. Thus, GF-2 may be a more promising and likely more economical anti-LOX ingredient for functional food and cosmeceutical applications. Furthermore, it is uncertain whether more potent anti-LOX peptides were missed from the set of 219 peptides, as the in silico tools used by Ding and coworkers [17] did not specifically screen for anti-LOX peptides during the process of narrowing down to the eight peptides. Meanwhile, enzyme kinetic experiments were not performed and the mode of LOX inhibition of the eight peptides was not investigated. Based on the antioxidant properties of the eight peptides, Ding and coworkers [17] speculated that the peptides may inhibit LOX by attenuating the formation of lipid hydroperoxides, which are substrates required for the LOX catalytic cycle [57]. 

**Figure 4:**
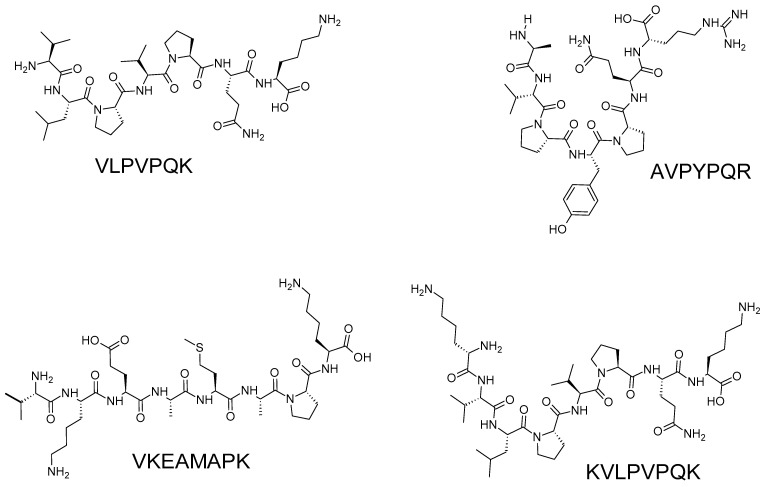
Anti-LOX peptides identified from a tryptic hydrolysate of β-casein [45]. The peptides were depicted using the ACD/ChemSketch freeware, as mentioned in the caption of Figure 3.

Four anti-LOX peptides of 7–8 residues were identified from a tryptic digest of β-casein [45,52] (Figure 4). Rival and coworkers [52] hypothesized that these four peptides inhibit LOX by acting as the preferred targets for carbon-centered radicals formed prior to the introduction of oxygen in LOX-catalyzed reactions. Their experimental data ruled out the possibility that the peptides acted as LOX inhibitors by forming enzyme-inhibitor complexes or by iron chelation [52]. Comparing the relative potency between the anti-LOX peptides derived from β-casein [52] and velvet antler blood [17] is challenging because the two studies used distinctly different LOX inhibition assays (spectrophotometric vs. rate of oxygen consumption) and due to the lack of an identical reference compound in their assays. Nonetheless, similar to Ding and coworkers [17], Rival and coworkers [52] found that synthetic peptides of the four sequences were less potent compared to the purified HPLC fractions containing the same peptides, and even less potent compared to unpurified tryptic hydrolysate of β-casein. The authors [52] proposed that the higher potency of the HPLC fractions may be due to the synergism between the peptides and the phosphate groups in the HPLC fractions. Alternatively, different conformations between the same peptide in the fraction and that in the synthetic form were also a possible contributing factor [52].

**Figure 5:**
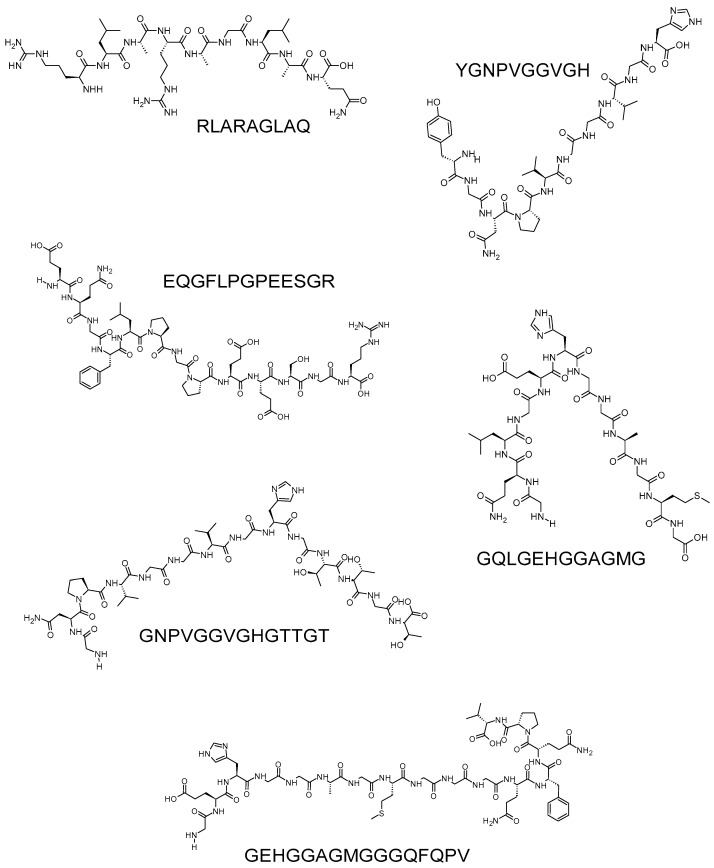
Anti-LOX peptides identified from the hydrolysates of millet protein fractions [6,55]. The peptides were depicted using the ACD/ChemSketch freeware, as mentioned in the caption of Figure 3.

A total of 6 anti-LOX peptides comprising 9–16 residues were identified from the millet grains [6,55] (Figure 5). The six peptides each contain at least one glycine residue, which accounts for 11% (RLARAGLAQ) to 50% (GQLGEHGGAGMG) of the amino acid composition of the peptides. The prevalence of glycine in the six peptides is consistent with the authors’ previous observation that glycine-rich (17–36%) peptide fractions derived from millet grains exhibited potent anti-inflammatory activities in vitro, including anti-LOX activity [38]. Among the six peptides, EQGFLPGPEESGR had the strongest anti-LOX activity (IC_50_ = 84.35 µg/mL), whereas RLARAGLAQ had the weakest (IC_50_ = 196.09 µg/mL) [6]. No well-established anti-inflammatory drugs or anti-LOX agents were tested in the study. A comparison of the peptides with a known active compound would have provided a more objective interpretation of the peptide potency. Notably, Złotek and coworkers [6] reported that RLARAGLAQ and GQLGEHGGAGMG were competitive inhibitors of LOX, whereas the other four peptides were non-competitive inhibitors. This is the only available report detailing the modes of inhibition, such as competitive or non-competitive, for protein hydrolysate-derived anti-LOX peptides of which we are aware.

Grancieri and coworkers [39] identified three putative anti-LOX peptides (HYGGPPGGCR, SPKDLALPPGALPPVQ, and TGPSPTAGPPAPGGGTH) from chia seed proteins subjected to simulated GI digestion (pepsin + pancreatin). In the study, anti-LOX capacity was expressed as ascorbic acid equivalents, and IC_50_ values were not reported. While all samples tested exhibited anti-LOX activity, hydrolysates of chia globulin, prolamin, and glutenin fractions were similarly potent, being stronger than the hydrolysate of total chia protein. Nonetheless, the authors did not proceed to synthesize the peptide sequences to verify their anti-LOX activity. The anti-LOX potential of the three peptides was predicted only based on their interactions with LOX in molecular docking simulation. The three peptides exhibited relatively negative binding free energies and lower inhibition constants (Ki) when compared to the pharmacological control Simvastatin, suggesting the potential of the peptides as LOX inhibitors [39]. Wet-lab validation of the anti-LOX activity of the three peptides is warranted in the future.

Several studies have reported the anti-LOX activity of protein hydrolysates and their fractions, but no further purification work was performed to unravel the specific peptide sequences responsible for the LOX inhibition. For example, the <3.5 kDa peptide fractions isolated from the hydrolysates of locust, mealworm larvae, and cricket protein isolates were shown to have anti-LOX activity, with IC_50_ values of 3.13, 3.82, and 6.95 µg/mL, respectively [40]. The study did not evaluate a known LOX inhibitor alongside the insect hydrolysate samples, although the authors attempted to compare the relative potency of their samples with those in the literature based on the reported IC_50_ values. Such comparisons should be treated with caution and may be misleading given possible differences in anti-LOX assay conditions or protocols used in different studies. 

Another interesting study compared the anti-LOX activity of two microbially produced feather keratin hydrolysates (hydrolyzed by *Streptomyces tanashiensis*-RCM-SSR-6 and *Bacillus* sp. RCM-SSR-102) with a hydrolysate generated using keratinase purified from *Bacillus* sp. RCM-SSR-102 [19,44]. Although all three hydrolysates exhibited 15-LOX inhibitory activity, the use of purified keratinase (IC_50_ = 95.40 µg/mL) seems to be a more effective strategy for producing a more potent hydrolysate when compared with the microbial hydrolysis (IC_50_ = 194–297 µg/mL) [19]. However, this study demonstrated that in addition to the commonly adopted enzymatic hydrolysis approach, anti-LOX protein hydrolysates can also be produced by microbial degradation. Microbial fermentation has advantages such as lower cost of peptide production compared with enzymatic hydrolysis, higher levels of protease activity contributed by all microbial proteases, and environmental friendliness. The successful use of microbial fermentation for producing antihypertensive peptides in the production of commercial dairy products has been reported [11].

A commercial salmon protein hydrolysate was reported to dose-dependently downregulate the expression of the arachidonate 12-LOX gene in both human gingival epithelial cells and human intestinal epithelial cells, along with other oxidative stress-related genes [58]. While the study presented interesting gene expression data in cell models, whether the downregulated 12-LOX gene expression could lead to reduced LOX activity in the cells is unknown. Neither were the peptides responsible for the downregulation of the LOX gene identified. The method used to produce the commercial hydrolysate was also not disclosed in the study.

## 5. Future Directions

In light of the research discussed above, we propose several potential research directions for the near future:

1. Protein hydrolysates and peptide fractions that have shown anti-LOX activity but whose constituent anti-LOX peptides have not yet been identified can be subjected to peptide identification as a next step. The identification of peptides with LOX inhibitory properties from protein hydrolysates remains scarce. Peptide identification followed by validation of their activities with synthetic peptides would further our understanding of the relationship between peptide structure and anti-LOX activity. In cases where a protein hydrolysate or partially purified peptide fractions exhibit stronger anti-LOX activity than the individual peptides, it will then be possible to test the hypothesis that the anti-LOX peptides act synergistically to account for the activity of the former. Anti-LOX protein hydrolysates and peptide fractions from feather keratins [19], fish scales [20], and insects [40] are promising candidates for the identification of anti-LOX peptides. Meanwhile, the three putative anti-LOX peptides (HYGGPPGGCR, SPKDLALPPGALPPVQ, and TGPSPTAGPPAPGGGTH) identified from chia seed proteins [39] that have not been validated for activity should proceed to synthesis and subsequence activity validation. In the long term, when a large dataset of anti-LOX peptides could be amassed, such information is useful for the development of a machine-learning-based anti-LOX peptide prediction server.
2. To date, none of the studies discussed above have reported protein hydrolysates and peptides that are more potent than established anti-LOX inhibitors. Whether this is an intrinsic property of the peptides as anti-LOX agents is unclear. Nevertheless, future research may consider exploring different biological sources and proteases for anti-LOX protein hydrolysate and peptide discovery. The diversity of samples from which anti-LOX protein hydrolysates and peptides have been produced (Table 1) suggests that anti-LOX capacities may be part of the protein hydrolysates and peptides of many other protein-rich raw materials, which could be explored more intensively in the future. In particular, the exploration of the anti-LOX properties of protein hydrolysates and peptides prepared from low-value agricultural wastes or by-products may contribute towards an efficient use of resources, a direction in line with Sustainable Development Goals (SDGs), e.g., SDG 12: Responsible Consumption and Production and SDG 3: Good Health and Well-being [59]. Meanwhile, more than 40 proteases of plant, animal, and bacterial origins are commercially available [11]. However, as shown in Table 1, fewer than 10 types of proteases have been used for the production of anti-LOX protein hydrolysates and peptides. Therefore, more enzymes should be tested in future. A promising strategy to be attempted in the future would be to systematically optimize the production of anti-LOX protein hydrolysates using the Response Surface Methodology (RSM) approach [60]. RSM can be applied to identify the optimal levels of parameters, such as enzyme type, enzyme:substrate ratio, hydrolysis time, and temperature, that maximize the anti-LOX activity of protein hydrolysates. The RSM approach has been used in previous studies to optimize the yield of bioactive protein hydrolysates and peptides [61,62].
3. Future evaluation of the anti-LOX capacity of all protein hydrolysates and peptides should include a well-established LOX inhibitor or an anti-inflammatory drug with anti-LOX capacity for comparison. This would allow for a more objective and convincing interpretation of anti-LOX potency, making it easier to compare, between studies, the anti-LOX potency of anti-LOX protein hydrolysates and peptides.
4. The in silico or cheminformatics strategy has not been sufficiently utilized to accelerate the discovery of anti-LOX peptides. In particular, molecular docking and molecular dynamics simulations can be more widely used to facilitate anti-LOX peptide discovery [63]. This can overcome the problem of a lack of in silico screening or prediction servers specifically designed for predicting anti-LOX peptides. This may also increase the chance of identifying peptides that inhibit LOX activity by forming a complex with LOX directly. On the other hand, if the sequences of major proteins in a sample targeted for anti-LOX peptide discovery are available in protein databases, e.g., UniProt Knowledgebase (https://www.uniprot.org/) [64], in silico hydrolysis can also be attempted to identify potential protease treatments for the sample. The BIOPEP-UWM server (https://biochemia.uwm.edu.pl/en/biopep-uwm-2/) is a free and user-friendly tool that allows users to perform enzymatic hydrolysis virtually using 33 proteases either singly or with up to 3 proteases simultaneously [4]. The server has been used to conduct in silico GI digestion of proteins in recent cheminformatic studies on bioactive peptides [65,66,67].
5. In view of the potential application of the anti-LOX protein hydrolysates and peptides as functional food ingredients, the effects of heat processing, pH conditions, and simulated GI digestion on the stability of such samples can also be investigated in future research [68]. Meanwhile, there are at least 20 peptidases and proteases in human blood [69]. Thus, the stability of anti-LOX protein hydrolysates and peptides in human blood is also of interest in the context of bioavailability. A simple reversed-phase high-performance liquid chromatography (RP-HPLC)-based assay to evaluate the stability of cytotoxic peptides in human blood has been reported previously [18], which could be applied to evaluate the stability of anti-LOX peptides in human blood.
6. The activity of the anti-LOX protein hydrolysates and peptides discussed above has yet to be demonstrated in biological models, both at the cellular and in vivo levels. Nair and Funk [70] developed a 96-well microplate fluorescence assay that can be used to screen samples for intracellular anti-LOX activity using mammalian Human Embryonic Kidney (HEK) 293 cells stably expressing 5-LOX, p12-LOX, and 15-LOX1 isoforms. Such cell-based anti-LOX assays can be used to confirm the potency of the anti-LOX protein hydrolysates and peptide fractions discussed above for further validation of bioactivity. This can serve as a further screen for promising candidates prior to proceeding to in vivo pharmacological evaluation, which is more costly and requires ethical approval.
7. The possibility of arachidonic acid being diverted to other pathways capable of producing proinflammatory mediators should not be overlooked when considering the use of LOX inhibitors. Cyclooxygenase (COX) can catalyze the production of prostaglandins from arachidonic acids [71,72]. Cytochrome P450 (CYP) enzymes (especially members of CYP4A and CYP4F subfamilies) can metabolize arachidonic acids into 20-hydroxyeicosatetraenoic acid and other bioactive lipids [73]. Such proinflammatory mediators are associated with inflammation and the development of diseases such as diabetes, cancer, and hypertension [73,74]. The search for multifunctional peptides that target COX, CYP, and LOXs is therefore an interesting research goal. It would be interesting to see whether these multifunctional peptides are more effective in reducing inflammation and preventing disease than single-function anti-LOX peptides.

## 6. Conclusions

Emerging evidence has demonstrated the ability of protein hydrolysates and peptides derived from various biological sources to act as LOX inhibitors. To date, only 18 anti-LOX peptide sequences have been documented. The majority of the research reviewed above is based on in vitro studies, with a lack of evidence from cellular and in vivo studies. Consequently, there are still significant gaps to be filled. In contrast to other bioactive peptides, such as anti-ACE and antioxidant peptides, a substantial amount of work is still needed to advance the discovery of anti-LOX peptides. This effort is essential to provide a solid foundation for their potential applications in the pharmaceutical, cosmetic, and food industries.

## Figures and Tables

**Table 1:** Proteolytic strategies employed for the production of anti-LOX protein hydrolysates and peptides.

Strategy	Raw material	Reference
Pepsin and pancreatin	Velvet antler blood	[17]
Pepsin and pancreatin	Chia seed total protein isolate; chia seed protein fractions (albumin, globulin, prolamin, and glutelin)	[39]
α-Amylase, pepsin, pancreatin, and bile extract	Mealworm larvae, locusts, and crickets	[40]
α-Amylase, pepsin, pancreatin, and bile extract	Millet protein fractions (albumin, globulin 7S, globulin 11S, prolamin, and glutelin)	[38]
Trypsin	β-casein	[45]
Neutral protease and keratinase	Fish diet consisting of white fish meal, fermented soybean meal, shrimp meal, and blood meal	[42]
Pepsin-soluble collagen extraction method	Scales of the milkfish (*Chanos chanos*)	[20]
Keratinolytic bacteria; purified keratinase enzyme	Poultry feather keratin wastes	[19]

**Table 2:** Anti-LOX peptides identified from hydrolyzed protein sources.

Peptide Sequence	Molecular Mass (Da)	Source	References
LFP	375.22 #	Velvet antler blood	[17]
FPH	399.19 #		
EHF	431.19 #		
VGYP	434.22 #		
FSAL	436.24 #		
LSQKFPK	846.51 #		
HHGGEFTPV	979.47 #		
LKECCDKPV	1147.55 #		
VLPVPQK	955.12	β-casein	[45]
AVPYPQR	956.03		
VKEAMAPK	1402.48		
KVLPVPQK	1070.14		
RLARAGLAQ	1210.27	Proso millet	[6,55]
YGNPVGGVGH	1485.59		
EQGFLPGPEESGR	955.12		
GQLGEHGGAGMG	956.03		
GNPVGGVGHGTTGT	1402.48		
GEHGGAGMGGGQFQPV	1070.14		

# Molecular mass was calculated from *m*/*z* data reported in the publication.

## Data Availability

Not applicable.
